# Frameshift indels introduced by genome editing can lead to in-frame exon skipping

**DOI:** 10.1371/journal.pone.0178700

**Published:** 2017-06-01

**Authors:** Simon Lalonde, Oliver A. Stone, Samuel Lessard, Adam Lavertu, Jessica Desjardins, Mélissa Beaudoin, Manuel Rivas, Didier Y. R. Stainier, Guillaume Lettre

**Affiliations:** 1 Montreal Heart Institute, Montreal, Quebec, Canada; 2 Department of Biochemistry & Biophysics, UCSF, San Francisco, California, United States of America; 3 Max Planck Institute for Heart and Lung Research, Bad Nauheim, Germany; 4 Faculty of Medicine, Université de Montréal, Montreal, Quebec, Canada; 5 Department of Biomedical Data Science, Stanford University, Stanford, California, United States of America; University of Florida, UNITED STATES

## Abstract

The introduction of frameshift indels by genome editing has emerged as a powerful technique to study the functions of uncharacterized genes in cell lines and model organisms. Such mutations should lead to mRNA degradation owing to nonsense-mediated mRNA decay or the production of severely truncated proteins. Here, we show that frameshift indels engineered by genome editing can also lead to skipping of “multiple of three nucleotides” exons. Such splicing events result in in-frame mRNA that may encode fully or partially functional proteins. We also characterize a segregating nonsense variant (rs2273865) located in a “multiple of three nucleotides” exon of *LGALS8* that increases exon skipping in human erythroblast samples. Our results highlight the potentially frequent contribution of exonic splicing regulatory elements and are important for the interpretation of negative results in genome editing experiments. Moreover, they may contribute to a better annotation of loss-of-function mutations in the human genome.

## Introduction

The success of genome-wide association studies (GWAS) in discovering DNA sequence variants associated with common human diseases and other complex traits has highlighted the challenge to characterize the functions of novel genes. Traditional strategies require gene knockdown using short-interfering RNA (siRNA) or over-expression with ectopic vectors in relevant cell types. Although powerful, these methods have their own caveats: siRNA can have off-target effects and over-expression can lead to non-physiological levels of gene expression. Fortunately, genome editing methodologies have emerged and are rapidly revolutionizing biological sciences [1]. Zinc finger nucleases and transcription activator-like effector nucleases (TALEN) were the initial tools of genome editing, but their use was limited by the complexity to target their nuclease activity to the loci of interest. More recently, the clustered regularly interspaced short palindromic repeats (CRISPR)-associated protein 9 (Cas9) system has become the preferred genome editing method because of its extreme simplicity and flexibility. This system relies on the synthetic design of single guide RNAs (sgRNAs) that direct the Cas9 nuclease to the loci of interest through DNA:RNA base pairing [2].

In its simplest and most popular application, CRISPR-Cas9 genome editing is used to introduce short frameshift insertion-deletions (indels) in exonic sequences to disrupt the reading frame of mRNA by introducing premature stop codons (PSC). These indels are created when the double-strand break generated by the Cas9 nuclease is repaired by non-homologous end joining (NHEJ) mechanisms. Under this experimental design, the assumption is that these mutated transcripts will be recognized and degraded by the nonsense-mediated mRNA decay (NMD) machinery [3], or will be translated into truncated non-functional proteins. Thus, frameshift indels generated by genome editing represent, in theory, a rapid and powerful approach to create loss-of-function (LoF) alleles to study gene function. This strategy has been employed to study genes of unknown function, and to perform genome-wide screens [4, 5].

In a recent study, Kapahnke and colleagues showed that indels introduced by CRISPR-Cas9 can lead to random splicing as opposed to mRNA degradation or protein truncation [6]. Specifically, they mutated the genes flotillin-1 (*FLOT1*) in HeLa cells and aspartylglucosaminidase (*AGA*) in HEK293T with CRISPR-Cas9. They found that these mutations could result in aberrant splicing of nearby exons, yielding truncated proteins or even proteins with in-frame insertions of novel amino acids. While conducting CRISPR-Cas9 experiments to characterize the phosphatase and actin regulator 1 (*PHACTR1*) gene, we also noted a new, although not random, splicing pattern for transcripts carrying frameshift indels. In this article, we present how splicing of “multiple of three nucleotides” exons that carry frameshift indels or nonsense variants can lead to shorter, but potentially functional, proteins. Together with results from Kapahnke et *al*. [6], our observations emphasize the importance to characterize the impact of CRISPR-Cas9-mediated potential LoF mutations at the RNA splicing and protein levels.

## Materials and methods

### Cell culture

Immortalized human aortic endothelial cells (teloHAEC) (ATCC, CRL-4052) were grown in vascular cell basal media (VCBM) (ATCC, PCS-100-030) supplemented with endothelial cell growth kit-VEGF (ATCC, PCS-100-041), 200 U/mL penicillin and 200 μg/mL of streptomycin (ThermoFisher, 15140122). Cells were maintained under a 5% CO_2_ atmosphere at 37°C and subcultured beyond reaching 90% confluency.

### Nuclease plasmid

pSpCas9(BB)-2A-Puro (PX459) V2.0 (https://www.addgene.org/62988) was used as a backbone for the generation of the pCas9-Neo nuclease expressing vector. Briefly, the puromycin resistance gene from pSpCas9(BB)-2A-Puro (PX459) V2.0 was replaced by a neomycin resistance (neoR) gene from a synthesized GeneArt Strings custom DNA fragment (ThermoFisher) containing the neoR sequence through restriction digest with EcoRI-HF (NEB, R3101) and ligation with T4 DNA ligase (NEB, M0202) to yield pCas9-Neo. Efficient protospacer sequences targeting the first three coding exons of the *PHACTR1* gene were selected using the Zhang Lab CRISPR Design website (http://crispr.mit.edu/). Three pairs of sgRNAs were consequently designed, specifically targeting exon 8 (sg-E8), exon 9 (sg-E9) and exon 10 (sg-E10) of *PHACTR1*. Oligonucleotides corresponding to the top and bottom strand of the sgRNAs were synthesized by IDT DNA, annealed and cloned into pCas9-Neo as previously described [7], generating three different bicistronic vector encoding the Cas9 nuclease and sg-E8, sg-E9 or sg-E10. Accurate cloning of neoR and sgRNAs from all generated plasmids were confirmed by Sanger sequencing.

### CRISPR-Cas9 genome editing

For CRISPR-Cas9 genome editing, cells were seeded at a density of 2x10^5^ cells per well in 6-well plates. A day after seeding, media was replaced with Opti-MEM I Reduced Serum media (ThermoFisher, 31985062) and cells were transfected with 0.5 or 1 μg of three differently targeting Cas9-sgRNA plasmids using the Lipofectamine 3000 Transfection Reagent (ThermoFisher, L3000001) over a 4-hour period, after which the transfection reaction was replaced with complete media. The next day, cells were passaged to monitor growth at sub-confluency levels. Two days post-transfection, cells were incubated with VCBM containing G418 at 200 μg/mL for positive selection of cells with nuclease plasmid integration. Antibiotic pressure was kept until all of non-transfected cells were dead (approximately 7 days). Genomic DNA of polyclonal populations was probed for confirmation of CRISPR-Cas9 indel events near expected cut-site by sequencing PCR products amplified with primers that anneal in introns flanking the targeted exons. Principally observed mutations occurring in these cell populations were determined using the TIDE (Tracking of Indels by DEcomposition) method [8]. A number of clonal cell lines were then generated by limiting dilution in order to recover clones with CRISPR-Cas9-mediated indels. Clonal cell lines were probed for specific genome reengineering events near the nuclease cut-site by Sanger sequencing of intron-intron PCR product from genomic DNA (gDNA).

### Intron-intron PCR for determination of CRISPR-Cas9-induced indels

Following clonal derivation of teloHAEC cell lines, gDNA was isolated using QuickExtract DNA Extraction Solution (Epicentre, QE0905) from 1x10^5^ cells. For intron-intron PCRs, 100 or 200 ng of gDNA was used as template for PCR reaction with the following primers: exon 8-targeted clones 5’-TGAGAAATTGCCGGACCAGT-3’ (forward) and 5’-ATAAACGCATCATCAACGCCA-3’ (reverse); exon 9-targeted clones 5’-CTACCCTGTCATAGGTGGTAATTTG-3’ (forward) and 5’-AAGGGCCTAAACAGGTGACTG-3’ (reverse); exon-10 targeted clones 5’-CGTGATGGATTCGGGTTAGAAA-3’ (forward) and 5’-TGGCATACTGGGACTAAGACT-3’ (reverse). gDNA from parental teloHAEC cells was used as control. Obtained PCR products were analysed by electrophoresis on a 1% agarose gel prior to Sanger sequencing.

### RNA extraction and quantitative PCR

In order to guarantee the reliability and reproduction of the results, RNA extraction, cDNA synthesis and qPCR experiments were conducted in accordance to the Minimum Information for Publication of Quantitative Real-Time PCR Experiments (MIQE) guidelines [9]. Total RNA was extracted using RNeasy Plus Mini kit (Qiagen) and analyzed with a RNA 6000 Nano kit (Agilent Technologies) to assess its concentration and integrity on an Agilent 2100 Bioanalyzer. Also, no contamination was found within RNA extracts as assessed by spectrophotometry using Take3 Micro-Volume plates (Biotek) with absorbance ratio of 260/280 nm greater or equal to 2.1 for all samples. cDNAs were then generated by reverse transcription from 1μg of total RNA (with RNA integrity number of 10 for all samples) using 1U of MultiScribe Reverse Transcriptase, 100mM dNTPS, 20U of RNase inhibitor and 1X Random Primers (Applied Biosystems, #4374966) in a 20 μL reaction volume. Reverse transcription reaction was carried in three steps: 10 minutes at 25°C, 120 minutes at 37°C and 5 minutes at 85°C. qPCR reactions were setup with 1.25 μL of cDNA (1/50 dilution based on dynamic range of previously done standard curve for all target genes), 5 μL of Platinum SYBR Green qPCR SuperMix-UDG (ThermoFisher) and 3.75 μL of primer pair mix at 0.8 μM each. qPCR reaction for each gene was performed in triplicates and carried in a CFX384 Touch Real-Time PCR Detection System (Bio-Rad, #1855485) with the following thermal profile: 2 minutes at 50°C, 15 minutes at 95°C and a three step cycle of 10 seconds at 95°C, 15 seconds at 55°C and 15 seconds at 72°C repeated 40 times. Following the amplification process, a melting curve analysis was performed to ensure the specificity of the amplified products. Also, resulting amplification products from previous qPCR standard curve experiments were run on 1% agarose gel and purified prior to Sanger sequencing in order to validate amplification of the desired target. To assert the absence of undesired contamination, qPCR reactions with no template controls for each gene were carried out simultaneously with no fluorescence detected. Cq values corresponding to the number of cycles to reach quantification threshold were determined with the CFX Manager 3.1 (Bio-Rad) software for all genes. Relative expression level for the *PHACTR1* gene was calculated by the ΔΔCT method (Schmittgen and Livak, 2008) normalized with the three reference genes Glyceraldehyde 3-phosphate dehydrogenase (*GAPDH*), Hypoxanthine Phosphoribosyltransferase 1 (*HPRT1*) and TATA-binding protein (*TBP*). Based on geNORM principles for accurate normalization of real-time quantitative RT-PCR data by geometric averaging of multiple internal control genes [10], a mean M value of 0.2618 was generated from the *GAPDH*, *HPRT1*, and *TBP* genes. The primers used were: human *PHACTR1* 5’-GAGGAGGAAGAGGAGGAGGA-3’ (forward, exon 12) and 5’-GGCCTGTTGCTGAGTTTGAT-3’ (reverse, exon 13); human *HPRT1* 5’-TGGCGTCGTGATTAGTGATG-3’ (forward) and 5’-CAGAGGGCTACAATGTGATGG-3’ (reverse); human *GAPDH* 5’-GACAGTCAGCCGCATCTTC-3’ (forward) and 5’-GCAACAATATCCACTTTACCAGAG-3’ (reverse); human *TBP* 5’-CGAATATAATCCCAAGCGGTTT-3’ (forward) and 5’-GTGGTTCGTGGCTCTCTTATCC-3’ (reverse). Primers for *PHACTR1* gene allow amplification of a region covering the end of exon 12 and beginning of exon 13, which is ubiquitous to all known *PHACTR1* transcripts.

### Transcript analysis by PCR

Total RNA from monoclonal cell lines was extracted and 1 μg was reverse transcribed as mentioned below. PCR reactions were performed with 1 μL of cDNA as template using 62.5 U of GoTaq Hot Start Polymerase (Promega, PRM5132) and 0.5 μM of primers flanking exon 6 or 7 and 11 of the *PHACTR1* gene. Primer sequences are as followed: exon 6 end 5’-GGTTGCCTCCAATGTCAAGT-3’ (forward), exon 7 end 5’- AGAGAGGCGGATGCATGTG-3’ (forward) and exon 11 beginning 5’-TCACTGGCAGACAAGGCAAT-3’ (reverse). cDNA from parental teloHAEC cells was used as control. Obtained PCR products were separated by electrophoresis on a 3% agarose gel prior to Sanger sequencing. A fraction of all visible and sufficiently isolated fragments were extracted from the gel by sampling the bands with a P1000 pipette tip. Band samples were re-amplified by PCR individually with the same conditions as mentioned above. Re-amplified PCR products were separated by electrophoresis on a 3% agarose gel and purified using QIAquick Gel Extraction Kit (Qiagen, # 28704) prior to sequencing. Sanger sequencing results from purified PCR products permitted the identification of sixteen individual *PHACTR1* transcripts that perfectly align with the reference sequence.

### Western blotting

Cell lysates from each monoclonal cell lines were prepared to evaluate PHACTR1 protein expression. Briefly, cells were lysed in RIPA buffer (50 mM Tris-HCl, pH 7.4; 1% NP-40; 0.25% Na-deoxycholate; 0.1% SDS; 150 mM NaCl) supplemented with protease inhibitor cocktail (Sigma), phosphatase inhibitor cocktail 2 and 3 (Sigma) and PMSF 1mM. Homogenized lysates were clarified by centrifugation to eliminate insoluble cell debris and solvated protein were recovered. Protein concentration was determined with a Pierce BCA Protein Assay Kit (ThermoFisher). Prior to SDS-Page, samples containing 25 μg of proteins were prepared in a reducing buffer containing 600 mM DTT and subsequently denatured at 95°C for 5 minutes. Samples were then loaded on 8% polyacrylamide gels, separated by electrophoresis in running buffer (25 mM Tris base; 192 mM glycine; 0.1% SDS) and transferred to nitrocellulose membranes in Towbin Buffer (25mM Tris base; 192 mM glycine, 20% (v/v) methanol). Blots were then incubated in blocking buffer (1x TBS, 0.1% Tween-20, 5% milk) for 2 hours at room temperature and probed with primary antibodies against PHACTR1 (1:1000; custom antibody generated by Biomatik) and GAPDH (1:10,000; Cell Signaling, #2118) at 4°C overnight. We confirmed the specificity of the anti-PHACTR1 antibody using siRNA knockdown experiments in teloHAEC. Afterwards, blots were incubated with horseradish peroxidase-conjugated anti-rabbit IgG (1:10,000; GE Healthcare, NA934) for 1 hour at room temperature. The immunoblotting signals were revealed with the Western Lightning Plus-ECLSubstrate (PerkinElmer, NEL105001EA) and visualized using the ChemiDoc Touch system (Bio-Rad, #1708370).

### Zebrafish experiments

All work was performed in accordance with national and international ethical guidelines. Tal effector-like nuclease (TALEN) pairs were designed to generate mutants in the zebrafish adhesion G protein-coupled receptor L4 (*adgrl4*) gene and each arm cloned using Golden Gate assembly into the pCS2^+^TAL3RR or pCS2^+^TAL3DD expression vectors. A TALEN targeting exon 2 of the *adgrl4* gene was generated with the following repeat variable diresidues (RVDs): NN HD HD NI NN NG HD NG NG NN NG HD NI HD HD HD NI NI NI NG and NN NG NG HD NG NN HD NI NG NG NG NI HD NI NN NI NG. One-cell stage embryos were injected with 30 pg total TALEN capped messenger RNA synthesized using the mMESSAGE mMACHINE kit (Ambion). Stable mutant alleles were identified using high-resolution melt analysis (HRMA) of PCR products generated with the following primers: zebrafish *adgrl4* 5’-GCTGGATCCATGCAGATTTCTTGAC-3’ (forward) and 5’- GCTGATTCCATTACCAGTGTACCCT-3’ (reverse). For analysis of *adgrl4* transcripts in wild type and mutant alleles, RNA and genomic DNA was isolated from individual embryos using TRIzol as previously described [11]. Briefly, individual 24 hours post fertilization (hpf) embryos were homogenised in TRIzol reagent, phases separated with chloroform and the aqueous phase stored at -80°C. For genomic DNA extraction, DNA was precipitated from the organic phase with ethanol, washed twice with 0.1 M sodium citrate in 10% ethanol and once with 75% ethanol. Genotyping was performed using the aforementioned *adgrl4* primers. For RNA extraction, the aqueous phase from 10–15 embryos per genotype (wild-type, heterozygous or mutant) was pooled, precipitated with isopropanol and washed with 70% ethanol. Genomic DNA was removed by DNase treatment for 1 hour at 37°C and total RNA was isolated using the Zymo Research RNA Clean & Concentrator kit. For PCR analysis, total RNA was reverse transcribed using the Superscript III First Strand Synthesis system and PCR was performed using primers amplifying a region of the *adgrl4* cDNA spanning from the 5’UTR to exon 8 of the gene. PCR products were visualized on a 1% agarose gel, and individual bands excised and cloned into the pGEM-T easy vector system for Sanger sequencing.

### Human erythroblast experiments

The cell culture protocol to proliferate and differentiate human CD34+ hematopoietic stem/progenitor cells (HSPCs) into erythroblasts has been described before [12, 13]. The protocols for RNA extraction and RNA-sequencing have also been described elsewhere [12]. Our RNAseq human erythroblast expression dataset is publicly available at NCBI GEO under accession number GSE90878. Genomic DNA extraction, genotyping on the Illumina HumanOmniExpress-12 v1.1 BeadChip array, and quality-control were performed as previously described [12]. Genotypes were imputed using the Michigan Imputation Server with the Haplotype Reference Consortium (HRC) panel (v. r1.1)[14].

Allelic imbalance (AI) was measured at each heterozygous genotype covered by RNA-sequencing in the 24 human erythroblast samples. Only SNPs directly genotyped or with high imputation quality (*R*^2^>0.6) were considered. Duplicated reads using Picard’s MarkDuplicates tool (v. 1.96) were removed. Each read was counted using the samtools (v 1.1) mpileup software and genome build hg19, and kept uniquely mapping reads using the -q 50 argument (mapping quality > 50) and sites with base quality >10. The analysis was further restricted to uniquely mapping sites as per the ENCODE 50-mer mappability track (score = 1) and removed sites showing mapping bias in simulations [15]. Sites with less than 30 overlapping reads were excluded. For a given heterozygous SNP, the statistical significance of AI was determined as the difference between the observed and expected ratio of “reference allele:alternate allele” with a binomial test. To account for read mapping bias, all reads overlapping all heterozygous SNPs in the RNA-sequencing dataset were summed and the expected ratio for each combination of alleles in each sample independently was calculated. For SNPs with high sequencing coverage, we down-sampled the number of reads that fell in the top 25^th^ coverage percentile so that the most covered sites do not bias the expected ratio [16]. A Bonferonni correction was used to account for the number of tests performed: the significance threshold for this AI experiment is α = 2x10^-5^.

## Results

**Fig 1:**
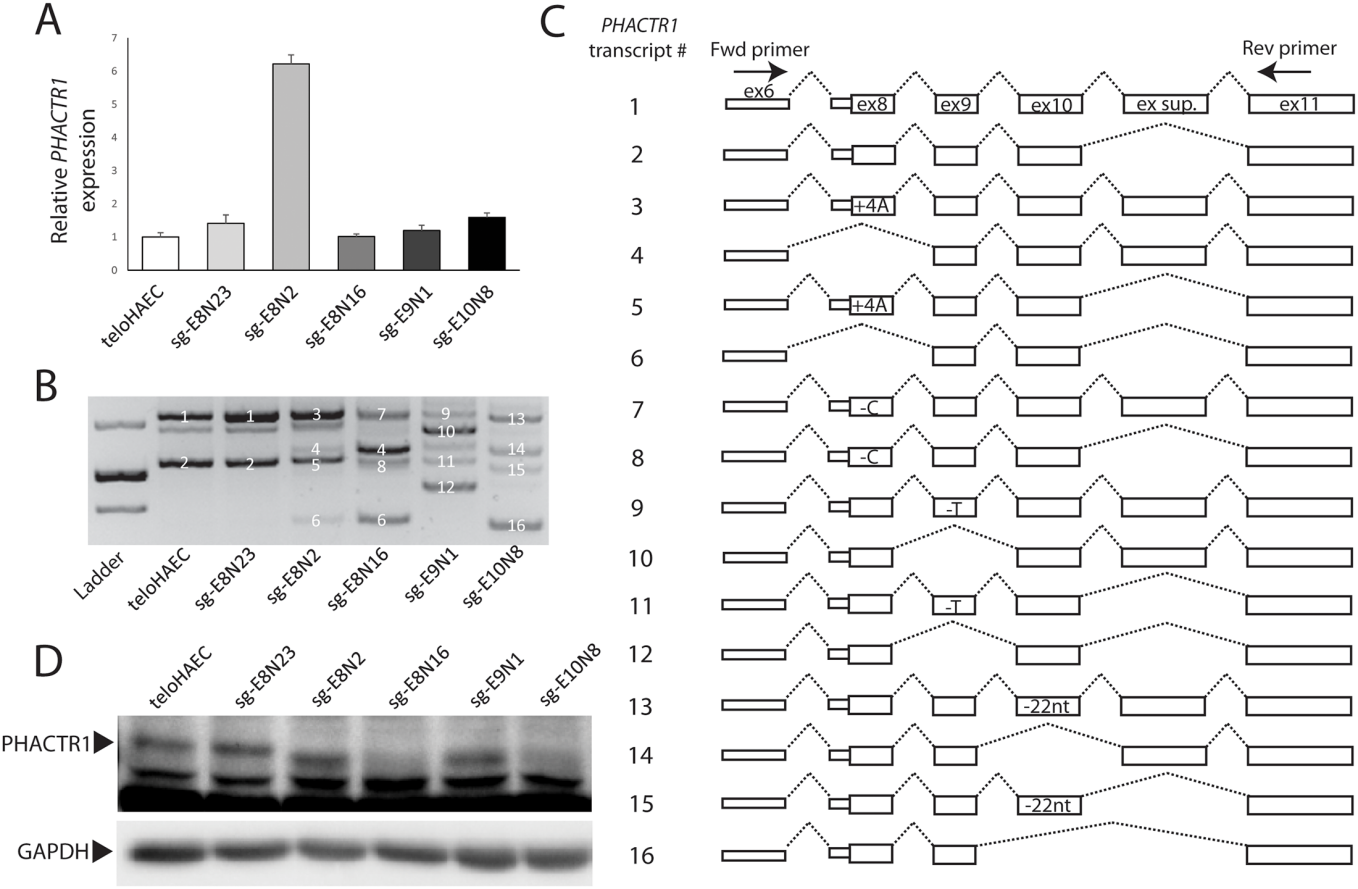
Frameshift indels cause in-frame exon skipping in *PHACTR1*. (**A**) *PHACTR1* expression levels measured by real-time qPCR in the parental teloHAEC cell line, an unedited clone (sg-E8N23), and clones with CRISPR-Cas9-generated frameshift indels in *PHACTR1* exon 8 (sg-E8N2 and sg-E8N16), exon 9 (sg-E9N1), and exon 10 (sg-E10N8). Data show mean and standard error of the mean from two biological replicates, done in triplicates. *PHACTR1* expression levels in sg-E8N2 is 6.2 fold greater than in the parental teloHAEC cell line (Student’s *t*-test *P* = 0.0033). (**B**) Agarose gel electrophoresis profile of the main *PHACTR1* isoforms detected in cDNA from teloHAEC cells, unedited clones, or clones with a frameshift indel. We assigned a transcript number to each of the *PHACTR1* isoform that we could Sanger sequence and align to the reference sequence. Unlabeled bands could not be assigned to *PHACTR1*. Bands in the molecular ladder correspond to 400, 500 and 700-bp. This gel is representative of three independent experiments. (**C**) Schematic diagram of all the *PHACTR1* isoforms that we identified in the different teloHAEC cell lines. Transcript numbers correspond to the bands (white numbers) in **B**. The PCR primers in exon 6 and 11 are depicted. For the isoforms expressed in edited clones, we added the corresponding nucleotide changes introduced by the frameshift indels. (**D**) Western blot of PHACTR1 in the parental, unedited and edited teloHAEC cells. The arrowhead indicates PHACTR1, lower bands are non-specific proteins recognized by the antibody. PHACTR1 is smaller in sg-E8N2, consistent with skipping of exon 8 or usage of an alternative in-frame start codon downstream of the frameshift indel. For sg-E9N1, the smaller PHACTR1 protein is consistent with skipping of exon 9. We could not detect PHACTR1 proteins in sg-E8N16 and sg-E10N8. We used GAPDH as loading control. This Western blot is representative of three independent experiments.

In an attempt to study the role of *PHACTR1*, a gene implicated in coronary artery disease by GWAS [17, 18], we used CRISPR-Cas9 to introduce frameshift indels in its first three translated exons in the teloHAEC cell line, and isolated four clones (S1 Table). We confirmed the homozygous state of these mutations by Sanger sequencing their genomic DNA: clones sg-E8N2 and sg-E8N16 harbour frameshift indels in exon 8, whereas sg-E9N1 and sg-E10N8 have frameshift indels in exons 9 and 10, respectively (S1 and S2 Tables). In teloHAEC, exons 6 and 7 are mutually exclusive untranslated *PHACTR1* exons. In Fig 1, we present results from the characterization of *PHACTR1* isoforms that include exon 6; we obtained consistent results with isoforms that start with exon 7 (S1 Fig). When we quantified expression levels in these edited cells, we observed that three of the clones had normal *PHACTR1* levels when compared to unedited cells, whereas one clone (sg-E8N16) with four extra A’s in exon 8 showed a ~6.2-fold increase (Fig 1A). The frameshift indels in exon 8 yield PSC that are located <200 nucleotides from the start codon, a distance that might be sufficient to escape NMD [19]. However, we anticipated that frameshift indels in exons 9 and 10, which are sufficiently distant from the start codon and the last exon, would reduce *PHACTR1* expression owing to NMD [19].

To understand the impact of these frameshift indels at the molecular level, we amplified, gel-purified, and Sanger-sequenced *PHACTR1* cDNA from these teloHAEC cells (Fig 1B). In the parental cells and an unedited clone (sg-E8N23), we identified two main isoforms that correspond to the known *PHACTR1* transcripts, with or without a supplementary exon located between exons 10 and 11 (Fig 1C, transcripts #1–2). For the four clones with frameshift indels, we found the same isoforms, carrying the corresponding mutations in the cDNA sequence. Strikingly, for each of these clones, we also identified two additional main isoforms generated by skipping the exon carrying the frameshift indel (Fig 1B and 1C). For instance, for clone sg-E10N8 that carries a 22-bp insertion in exon 10, we found one transcript without exon 10 (transcript #14 in Fig 1B and 1C) and one without exon 10 and the supplementary exon (transcript #16 in Fig 1B and 1C). Because exons 8, 9, 10, and the supplementary exon of *PHACTR1* are all a multiple of three nucleotides in length, their skipping yields in-frame transcripts that could be translated into slightly shorter proteins. By Western blot, we observed smaller PHACTR1 proteins in sg-E8N2 and sg-E9N1 (Fig 1D). Because of exon skipping, these proteins are predicted to lack, respectively, 53 and 27 amino acids, and we are currently comparing their functions with wild-type PHACTR1. We did not detect PHACTR1 in sg-E8N16 and sg-E10N8 by Western blot (Fig 1D), but we need to perform additional tests to determine if these clones have any residual PHACTR1 functions.

**Fig 2:**
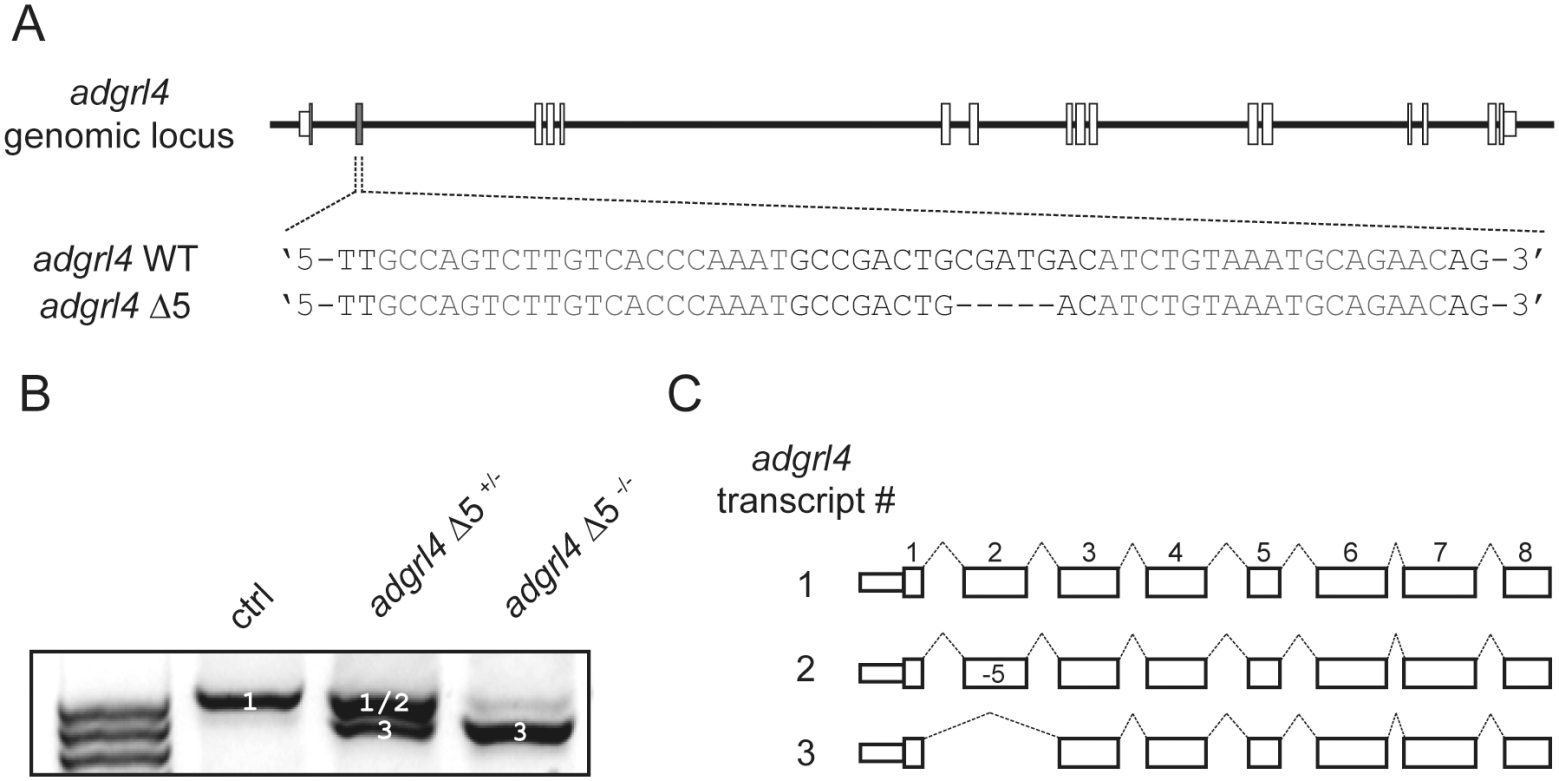
Frameshift indel can lead to exon skipping in the zebrafish *adgrl4* gene. (**A**) *adgrl4* genomic locus, TALEN target site in exon 2 of the *adgrl4* gene and a stable mutant line (Δ5) that was analyzed. (**B**) PCR analysis of *adgrl4* mRNA transcripts in 10 to 15 pooled embryo samples from control (ctrl), *adgrl4* Δ5^+/-^, and *adgrl4* Δ5^-/-^ fishes. (**C**) Schematic representation of the different transcripts recovered from the bands (white numbers) in **B**.

These results illustrate how cells can bypass the consequences of frameshift indels by generating, through the skipping of exons, an in-frame mRNA that can be translated into a shorter but potentially functional protein. We documented another example of this phenomenon when targeting the zebrafish *adgrl4* gene, which has been implicated in vascular development [20]. Using TALEN, we introduced a frameshift indel (Δ5) into exon 2 —a “multiple of three nucleotides” exon—of the *adgrl4* gene (Fig 2A). As with the *PHACTR1* CRISPR-Cas9 mutations, the Δ5 frameshift allele led to efficient skipping of *adgrl4* exon 2 (*adgrl4* Δ5 ^-/-^ in Fig 2B and 2C). Skipping of exon 2 results in an in-frame transcript encoding a predicted protein that is 44 amino acids shorter than full-length Adgrl4, and lacks one of three extracellular EGF-like repeats.

**Fig 3:**
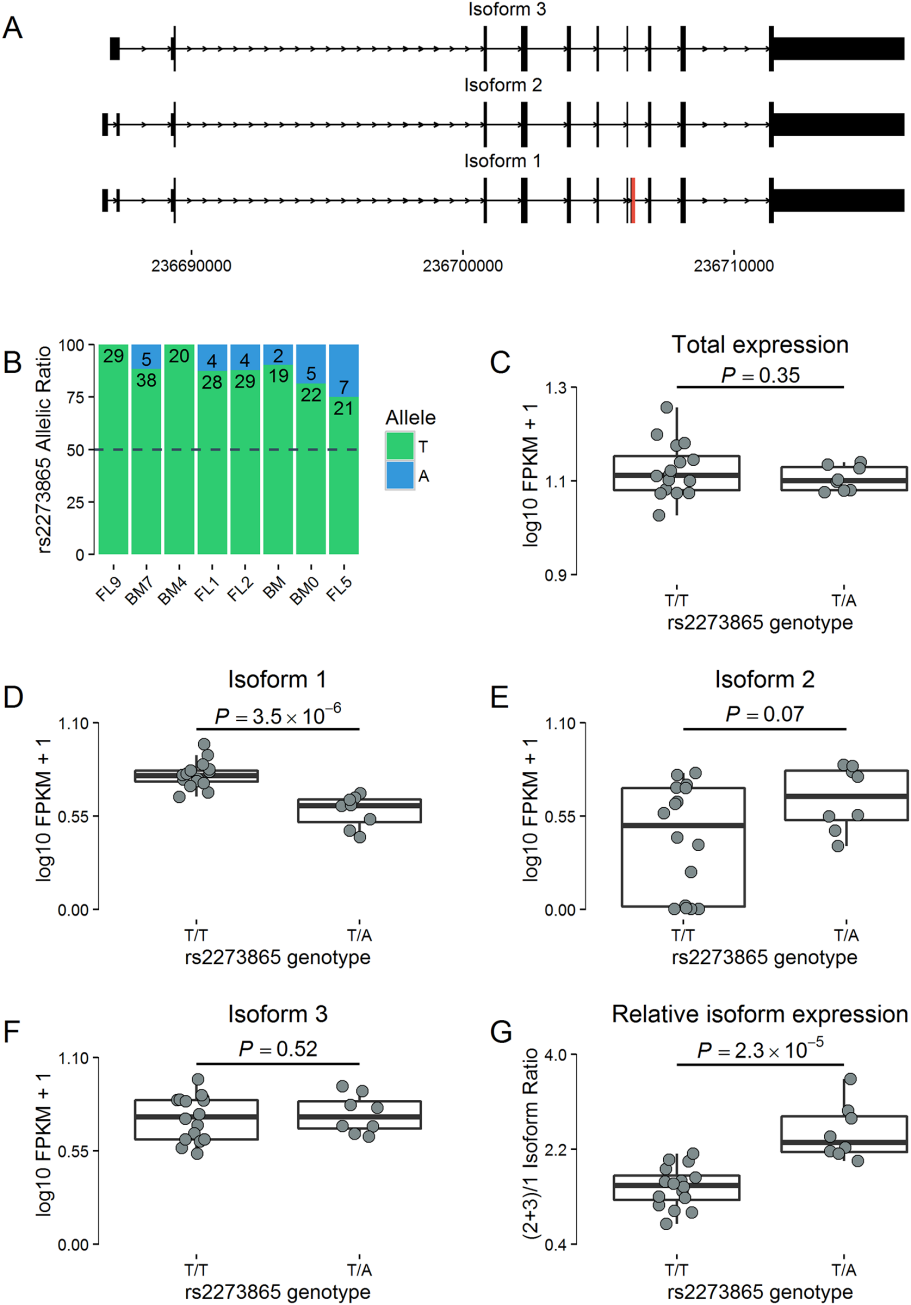
Exon skipping in *LGALS8* in human erythroblasts. (**A**) In *in vitro* differentiated human erythroblasts, three *LGALS8* mRNA isoforms are expressed. Isoform 1 includes the “multiple of three nucleotides” exon 9 (in red, 126-bp), whereas isoforms 2 and 3 do not. The nonsense variant rs2273865 (p.Leu212Ter) is located in exon 9. At this variant, the minor A-allele has a frequency of 3.5% in populations of European ancestry (ExAC). (**B**) Eight erythroblast samples are heterozygous at rs2273865 and show strong allelic imbalance (binomial *P*<0.05 for all samples). Numbers in the bars indicate the numbers of reads carrying the T (green) or A (blue) allele. Differential expression of total *LGALS8* (**C**), isoform 1 (**D**), isoform 2 (**E**), and isoform 3 (**F**) between erythroblast samples homozygous TT (n = 16) and heterozygous AT (n = 8) at rs2273865. No samples homozygous for the minor allele (AA) were available. (**G**) The ratio of *LGALS8* transcripts without exon 9 over transcripts with exon 9 is higher in heterozygous AT than in homozygous TT erythroblast samples. *P*-values are calculated by linear regression correcting for cell developmental stage.

The two previous examples illustrate how protein-truncating mutations introduced by genome editing in cell lines or model organisms can favour skipping of “multiple of three nucleotides” exons over NMD-mediated transcript degradation. We next asked if we could extend these observations to gene expression phenotypes in human samples. In a dataset of 24 human erythroblast samples with full transcriptomic and genetic information available [12], we detected strong allelic imbalance (AI) for a nonsense variant (rs2273865_T/A, p.Leu212Ter, minor A-allele frequency = 3.5% in Europeans from ExAC) in *LGALS8*, which encodes a glycan-binding protein (Fig 3A and 3B). Three *LGALS8* isoforms are expressed in erythroblasts: isoform 1 that contains exon 9 where rs2273865 resides, and isoforms 2 and 3 without exon 9 (Fig 3A). Despite the AI effect, total *LGALS8* expression levels between homozygous TT and heterozygous AT samples were similar (Fig 3C, we did not have homozygous AA samples in our collection). However, we found that the levels of isoform 1 (with exon 9) were lower in heterozygous samples but that this reduced expression was compensated by increase synthesis of isoform 2 (without exon 9) (Fig 3D and 3E). The levels of isoform 3 were not different between homozygous and heterozygous erythroblast samples (Fig 3F). When we considered the ratio of *LGALS8* transcripts without exon 9 (isoforms #2 and #3) over those with exon 9 (isoform #1), we detected increased exon 9 skipping in heterozygous AT samples (Fig 3G). Because *LGALS8* exon 9 is a “multiple of three nucleotides” exon (126-bp), its skipping yields an in-frame transcript which may encode a protein with complete or partial activity that may rescue the nonsense variant. This result is completely consistent with our genome editing findings for *PHACTR1* in teloHAEC and *adgrl4* in zebrafish, and indicates that the skipping of “multiple of three nucleotides” exons that carry nonsense or frameshift indel variants can also influence human phenotypes.

## Discussion

Genome editing approaches, and in particular the CRISPR-Cas9 method, provide a powerful and flexible framework to study the function of genes in their cellular context. Generally, the absence of phenotypes caused by these engineered frameshift indels, which are readily labeled as strong LoF mutations, automatically indicates that the genes play no role in the biological assays under investigation. However, our results, combined with observations from another study [6], indicate that this conclusion is often premature. Indeed, frameshift indels or nonsense variants can influence splicing patterns, leading to shorter proteins with a possibly maintained partial function(s). Kapahnke and colleagues determined that CRISPR-Cas9-mediated frameshift indels can randomly modulate the splicing pattern of nearby exons, resulting in shorter proteins, or proteins with new amino acid sequences [6]. By focusing on exons that are multiple of three nucleotides in *PHACTR1*, *adgrl4*, and *LGALS8*, we noticed that exon skipping is not random, but always involved the exon that carries the mutation: this results in shorter, in-frame transcripts. In contrast to Kapahnke et *al*. [6], we did not find transcripts with new amino acid sequences, but this might be explained by differences in exon sizes for each of the different genes studied. Our additional contributions include the confirmation that this exon skipping can occur *in vivo* (zebrafish) and with TALEN-mediated mutations. Furthermore, we have shown using an expression quantitative trait loci (eQTL) dataset of human erythroblasts that DNA sequence variation that introduces a PSC in a “multiple of three nucleotides” exon can lead to its precise skipping in order to avoid NMD.

Our results are important for at least three reasons. First, they provide one possible explanation for silent frameshift indels introduced by genome editing. Indeed, even if frameshift indels are predicted to trigger NMD or to result in truncated non-functional proteins, our data illustrate an alternative, potentially phenotypically neutral route: skipping of “multiple of three nucleotides” exons carrying the frameshift indels. This might be particularly important in the interpretation of negative results from genome-wide CRISPR-Cas9 screens, where splicing patterns of single genes are usually not monitored. A simple solution is to target “non-multiple of three nucleotides” exons: even if NMD is bypassed, exon skipping would result in out-of-frame transcripts. Second, by considering the possibility that frameshift indels in “multiple of three nucleotides” exons may lead to exon skipping and in-frame transcripts, we might improve the annotation of LoF alleles in the human genome. There is a tremendous interest in using segregating LoF alleles to assign functions to genes by phenotyping individuals that carry these mutations [21–23]. Although frameshift indels located away from the start codon and the last exon are usually assigned as strong LoF alleles, our analyses indicate that some of them located in “multiple of three nucleotides” exons might be hypomorphic or even silent.

Finally, beyond essential splice sites at exon-intron boundaries, the use of minigenes has helped uncover many types of exonic and intronic regulatory elements that contribute to splice site selection [24]. The density of such elements in alternative exons can be surprisingly high [25, 26]. Despite this progress, it has been notoriously difficult to identify and validate which sequences—either exonic or intronic—control splicing patterns in transcripts produced endogenously. Genome editing will likely emerge as a powerful tool to dissect the rules that control splicing decisions in natural chromatin context [27]. High-throughput experiments that combine genome editing with transcriptomic analyses may yield sufficient molecular insights to design more accurate bioinformatic tools to predict the impact of specific DNA sequence variants on splicing.

## Supporting information

S1 Table Annotation of *PHACTR1* exons. (DOCX)

S2 Table Annotation of *PHACTR1* frameshift indels generated in this project in teloHAEC cells with CRISPR-Cas9. (DOCX)

S1 Fig Frameshift indels cause in-frame exon skipping in *PHACTR1*. (DOCX)
