# Supplementary material for: Frameshift indels introduced by genome editing can lead to in-frame exon skipping
Source: PLoS One. 2017 Jun 1;12(6):e0178700. doi: 10.1371/journal.pone.0178700 (PMC5453576; doi:10.1371/journal.pone.0178700)
Supplement: S1 Table — (DOCX) [file pone.0178700.s001.docx]

**S1 Table.** Annotation of *PHACTR1* exons. Coordinates are on the UCSC Genome Browser build GRCh38/hg38. bp, basepairs; aa, amino acids.

| **Exon** | **Chromosome** | **Start position** | **End position** | **Size (bp, (aa))** |
| --- | --- | --- | --- | --- |
| 6 | 6 | chr6:12,957,404 | chr6:12,957,545 | 141 |
| 7 | 6 | chr6:12,957,982 | chr6:12,958,038 | 56 |
| 8 | 6 | chr6:13,053,365 | chr6:13,053,530 | 165 (46-47) |
| 9 | 6 | chr6:13,160,204 | chr6:13,160,285 | 81 (27) |
| 10 | 6 | chr6:13,182,519 | chr6:13,182,687 | 168 (56) |
| Supplementary | 6 | chr6:13,184,799 | chr6:13,185,006 | 207 (69) |
| 11 | 6 | chr6:13,205,815 | chr6:13,206,137 | 322 |
