# Supplementary material for: Frameshift indels introduced by genome editing can lead to in-frame exon skipping
Source: PLoS One. 2017 Jun 1;12(6):e0178700. doi: 10.1371/journal.pone.0178700 (PMC5453576; doi:10.1371/journal.pone.0178700)
Supplement: S2 Table — (DOCX) [file pone.0178700.s002.docx]

**S2 Table.** Annotation of *PHACTR1* frameshift indels generated in this project in teloHAEC cells with CRISPR-Cas9. Coordinates are on UCSC Genome Browser build GRCh38/hg38. NA, not applicable; PSC: premature stop codon.

| **Clone** | **Frameshift indel** | **Coordinates (Chr:Pos)** | **Predicted effect** |
| --- | --- | --- | --- |
| wild-type (WT) | NA | NA | NA |
| sg-E2N23 | NA | NA | NA |
| sg-E8N2 | Insertion of 4 A’s in exon 8 | chr6:13,053,435 | This indel introduces a premature stop codon in exon 8, 60 nucleotides from the start codon. Could escape nonsense-mediated mRNA decay. |
| sg-E8N16 | Deletion of 1 C in exon 8 | chr6:13,053,435 | This indel introduces a premature stop codon in exon 9, 159 nucleotides from the start codon. Could escape nonsense-mediated mRNA decay. |
| sg-E9N1 | Deletion of 1 T in exon 9 | chr6:13,160,264 | This indel introduces a PSC in exon 10 located 255 nucleotides downstream of the start codon. Unlikely to escape nonsense-mediated mRNA decay. |
| sg-E10N8 | Deletion of 22 nucleotides in exon 10 | chr6:13,182,606 | This indel introduces a PSC in the supplementary exon located 495 nucleotides downstream of the start codon. Unlikely to escape nonsense-mediated mRNA decay. |
|  |  |  | If the supplementary exon is not expressed, this indel introduces a PSC in exon 11 located 381 nucleotides downstream of the start codon. Unlikely to escape nonsense-mediated mRNA decay. |
