# Supplementary material for: Frameshift indels introduced by genome editing can lead to in-frame exon skipping
Source: PLoS One. 2017 Jun 1;12(6):e0178700. doi: 10.1371/journal.pone.0178700 (PMC5453576; doi:10.1371/journal.pone.0178700)

**S1 Figure.** Frameshift indels cause in-frame exon skipping in *PHACTR1*. (**A**) These results are analogous to those presented in **Fig 1**, except that they capture *PHACTR1* isoforms that include exon 7 (exons 6 and 7 are mutually exclusive). (**A**) Agarose gel electrophoresis profile of the main *PHACTR1* isoforms detected in cDNA from teloHAEC cells, unedited clones (sg-E8N23), or clones with a frameshift indel (exon 8 (sg-E8N2 and sg-E8N16), exon 9 (sg-E9N1), and exon 10 (sg-E10N8)). We assigned a transcript number to each of the *PHACTR1* isoform that we could Sanger sequence and align to the reference sequence. Unlabeled bands could not be assigned to *PHACTR1*. (**B**) Schematic diagram of all the *PHACTR1* isoforms that we identified in the different teloHAEC cell lines. Transcript numbers correspond to the bands (white numbers) in **A**. The PCR primers in exon 7 and 11 are depicted. For the isoforms expressed in edited clones, we added the corresponding nucleotide change introduced by the frameshift indels.


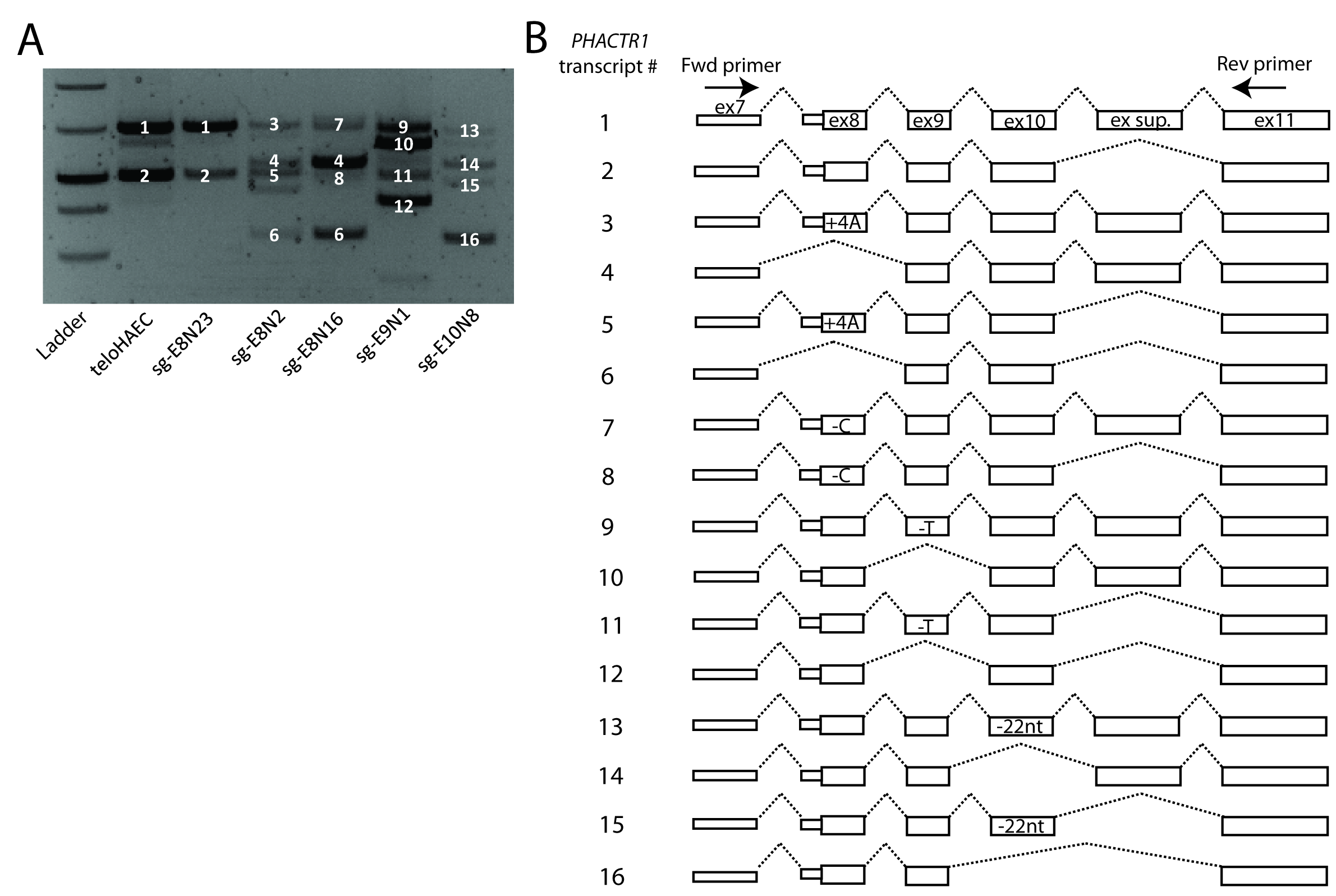

Supplement: S1 Fig — (DOCX) [file pone.0178700.s003.docx]
